# Supplementary material for: scTWAS: a powerful statistical framework for single-cell transcriptome-wide association studies
Source: Nat Commun. 2026 Mar 12;17:3853. doi: 10.1038/s41467-026-70374-7 (PMC13121454; doi:10.1038/s41467-026-70374-7)
Supplement: Supplementary file 1 — Supplementary Information [file 41467_2026_70374_MOESM1_ESM.pdf]

Supplementary Information for "scTWAS: A powerful statistical framework for single-cell transcriptome-wide association studies"

Zhaotong Lin, Chang Su

Contents

1 Supplementary Methods **2**

1.1 A detailed description of the IRLS algorithm 2

1.2 Estimation of hidden batch effects in the ROSMAP single-cell data . . . 3

1.3 Additional simulation results 3

1.3.1 Out-of-sample LD 3

1.3.2 Pleiotropy effects of *cis*-SNPs 4

2 Supplementary Tables **5**

3 Supplementary Figures **6**

1 Supplementary Methods

1.1 A detailed description of the IRLS algorithm

In this section, we provide implementation details of the iteratively reweighted least squares (IRLS) algorithm used to estimate the genetic effects on gene expression in our scTWAS framework. Specifically, we describe three key components: (1) preprocessing with `sctransform`, (2) parameter configuration in penalized regression using `cv.glmnet`, and (3) truncation of regression weights to ensure numerical stability.

Algorithm 1 in the main text requires a vector of initial weights $\boldsymbol{\omega}^{(0)}$ and an overdispersion parameter θ as input for a given gene g. In practice, we apply the `SCTransform()` function in the R package `Seurat` to the pseudo-bulk count matrix constructed from single-cell data of a specific cell type, with all genes and individuals to be analyzed. This function fits a regularized negative binomial model, regressing out technical covariates such as sequencing depth [1]. We then extract the estimated intercept β_{0g} and overdispersion parameter θ_g for gene g using the `SCTResults()` function. The gene-level baseline mean expression (with no genetic effects) for individual i is computed as $s_i\mu_{ig}^{(0)}$ for $\mu_{ig}^{(0)} = \exp(\beta_{0g})$, where s_i is the sequencing depth for individual i. The corresponding variance is given by ${\sigma_{ig}^{(0)}}^2 = {\mu_{ig}^{(0)}}^2/\theta_g$ by the definition of the overdispersion parameter [1, 2]. The variance of the observed counts is then given by $s_i\mu_{ig}^{(0)} + s_i^2{\sigma_{ig}^{(0)}}^2$ and further truncated by a lower bound `min_var` returned by `SCTResults()`. Finally, the initial IRLS weights is calculated as $\omega_i^{(0)} = 1/(s_i\mu_{ig}^{(0)} + s_i^2{\sigma_{ig}^{(0)}}^2)$.

To fit the gene expression prediction model, we use elastic net regression via the `cv.glmnet` function in R (line 4 in Algorithm 1). We set `penalty.factor` to be a vector of ones for all *cis*-SNPs and zeros for covariates, ensuring that penalization is applied only to genetic predictors while leaving covariates unpenalized. We set `intercept = FALSE` as we incorporate the intercept term in the covariate $\mathbf{C}$, and `standardize = FALSE` since the input features (genotype dosages) have already been standardized. The mixing parameter α for elastic net is fixed at 0.5. The tuning parameter λ is selected through

five-fold cross-validation.

Finally, during the IRLS updates, estimated weights can become extreme, leading to unstable algorithm performance. To mitigate this, we apply truncation to the weights used in the weighted least squares step (line 7 in Algorithm 1) by ensuring that the variance remains above the `min_var` value returned by `SCTResults()`.

1.2 Estimation of hidden batch effects in the ROSMAP single-cell data

We performed principal component analysis (PCA) on sctransform-normalized pseudo-bulk gene expression in microglia, and found a clear separation between groups of samples by the value of PC 1 (Supplementary Figure 20a). We found that the separation can be explained by technical batch effects: samples from a subset of batches (B4, B5, and MAP) are separated from samples from other batches (Supplementary Figure 19), suggesting that PC 1 may capture hidden batch effects. Hence, we applied k-means clustering with the number of clusters equal to 2 on PC 1 and constructed a binary covariate to represent the hidden batch effect. Moreover, this covariate also separates samples in other cell types (Supplementary Figure 20b-d), suggesting that the hidden batch effect is universal across cell types. Consequently, we choose to include it as a covariate in the Stage 1 prediction models for all cell types.

1.3 Additional simulation results

1.3.1 Out-of-sample LD

Let $\mathbf{V}$ be the LD matrix estimated from OneK1K. As described in Section "Two-sample TWAS with GWAS summary data" in the main text, the GWAS summary statistics are simulated with $\mathbf{V}$. To mimic LD mismatch encountered when using an external reference panel, we generate the LD matrix $\mathbf{V}_{\text{ref}}$ (from the same ancestry) by sampling from

$$\mathbf{S} = \frac{1}{\nu}\mathbf{W}, \quad \mathbf{W} \sim \text{Wishart}(\nu, \mathbf{V}_\lambda), \quad \mathbf{V}_\lambda = (1 - 10^{-3})\mathbf{V} + 10^{-3}\mathbf{I} \tag{1}$$

with $\nu = 2000$ representing the effective LD reference size. We then set $\mathbf{V}_{\text{ref}} = \text{cov2cor}(\mathbf{S})$, and use $\mathbf{V}_{\text{ref}}$ in TWAS Stage 2. The TWAS test statistics is $\mathbf{z}_{\text{TWAS}} = \frac{\widehat{\boldsymbol{\beta}}' \mathbf{z}_{\text{GWAS}}}{\sqrt{\widehat{\boldsymbol{\beta}}' \mathbf{V}_{\text{ref}} \widehat{\boldsymbol{\beta}}}}$, where $\widehat{\boldsymbol{\beta}}$ denotes the estimated Stage 1 eQTL weights and $\mathbf{z}_{\text{GWAS}} = \widehat{\boldsymbol{\beta}}_{\text{GWAS}} / \text{SE}(\widehat{\boldsymbol{\beta}}_{\text{GWAS}})$ is the vector of GWAS Z-scores generated from Eq. (13) in the main text. As shown in Fig. 7, results are almost identical to those obtained with the true in-sample LD matrix $\mathbf{V}$ (Figure 1c in the main text).

1.3.2 Pleiotropy effects of *cis*-SNPs

To allow *cis*-SNPs to have direct effects on the GWAS trait independent of gene expression, we simulated the true joint SNP effect on the GWAS trait $\mathbf{b}_{\text{GWAS}} = \gamma \boldsymbol{\beta} + \boldsymbol{\alpha}$, where $\boldsymbol{\beta}$ is the eQTL effects on the gene expression, γ is the scaling factor to control h_g^2 as in the main text, and $\boldsymbol{\alpha}$ is the horizontal pleiotropy effect. Specifically, we randomly selected 2 out of the 10 causal SNPs of gene *AAK1* to have non-zero elements in $\boldsymbol{\alpha}$, and their effect sizes were scaled such that they explained around $h^2_{\text{pleio}} = k \cdot \frac{0.2}{180}$ of the trait variance, where $k \in \{0.5, 1\}$. As shown in Fig. 8, under horizontal pleiotropy, all three methods showed inflated type-I errors, and the inflation became more severe when k increased from 0.5 to 1 (reflecting stronger pleiotropic effects). When type-I error is estimated based on Stage 2 p-values (Fig. 8a, left column), the inflation is similar across methods, suggesting that all methods are comparably susceptible to horizontal pleiotropy. Although scTWAS appears more inflated when significant discoveries are further conditioned on having a predictive Stage 1 model (Fig. 8b, left column), this reflects that scTWAS more often produces a predictive Stage 1 model, rather than more inflated Stage 2 p-values due to horizontal pleiotropy. We also applied LDA-Egger [3], a robust TWAS method, to the pleiotropic scenarios. As shown in Fig. 9, type-I errors were reduced for all three methods, but power was also markedly reduced. Overall, these findings indicate that all three TWAS approaches are vulnerable to horizontal pleiotropy, consistent with the well-known sensitivity of standard TWAS to pleiotropic effects [4]. Developing pleiotropy-robust extensions of scTWAS is an important direction for future work.

2 Supplementary Tables

(a) Non-null Stage 1 and null Stage 2.

	$CD4_{NC}$	$CD8_{NC}$	B_{IN}	$Mono_{C}$	NK_{R}	Plasma
NA-TWAS	0.038	0.037	0.023	0.01	0.003	0.003
AN-TWAS	0.031	0.046	0.043	0.015	0.006	0.001
scTWAS	0.042	0.046	0.048	0.027	0.009	0.005

(b) Null Stage 1 and null Stage 2.

	$CD4_{NC}$	$CD8_{NC}$	B_{IN}	$Mono_{C}$	NK_{R}	Plasma
NA-TWAS	0.001	0.001	0.001	0.001	0.001	0.001
AN-TWAS	0	0.003	0.002	0.001	0.001	0.002
scTWAS	0	0.001	0.002	0	0.002	0

Table 1. **Empirical type-I error rates of gene-trait association tests in one-sample individual-level simulations.** (a) Gene expression was simulated as a function of *cis*-SNPs in Stage 1, and phenotype was simulated to be independent of gene expression in Stage 2. (b) Gene expression was independent of *cis*-SNPs in Stage 1, and phenotype was also independent of gene expression in Stage 2. A gene-trait association is considered significant if the Stage 1 p-value and Stage 2 p-value are less than 0.05.

3 Supplementary Figures

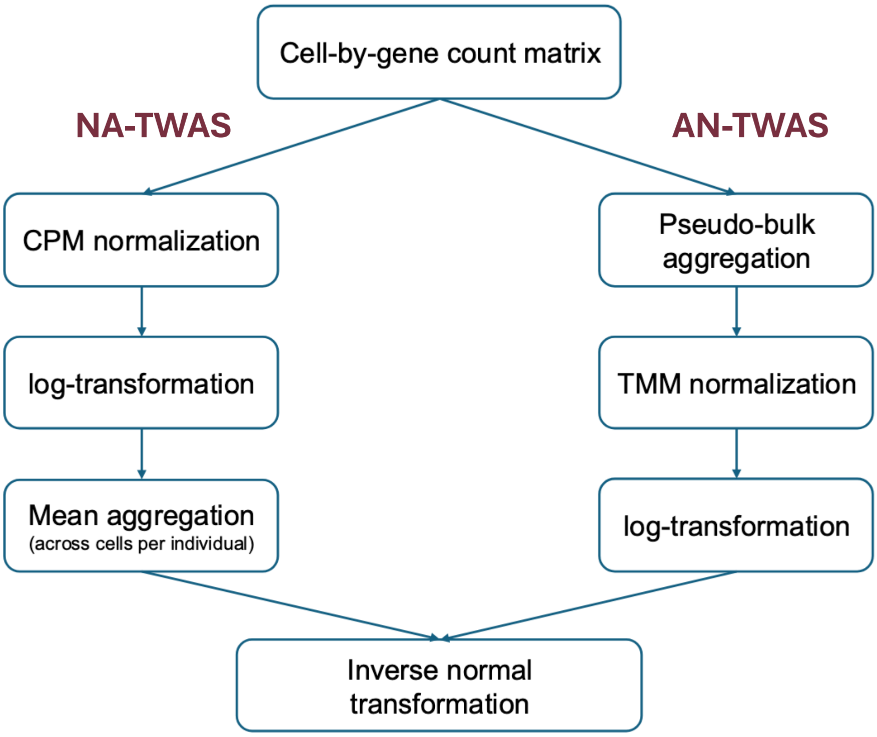

Figure 1. **Normalization steps used in NA-TWAS [5] and AN-TWAS [6].** The flowchart illustrates the preprocessing pipelines applied to single-cell or single-nucleus RNA-seq data before applying the traditional TWAS framework in NA-TWAS (left) and AN-TWAS (right).

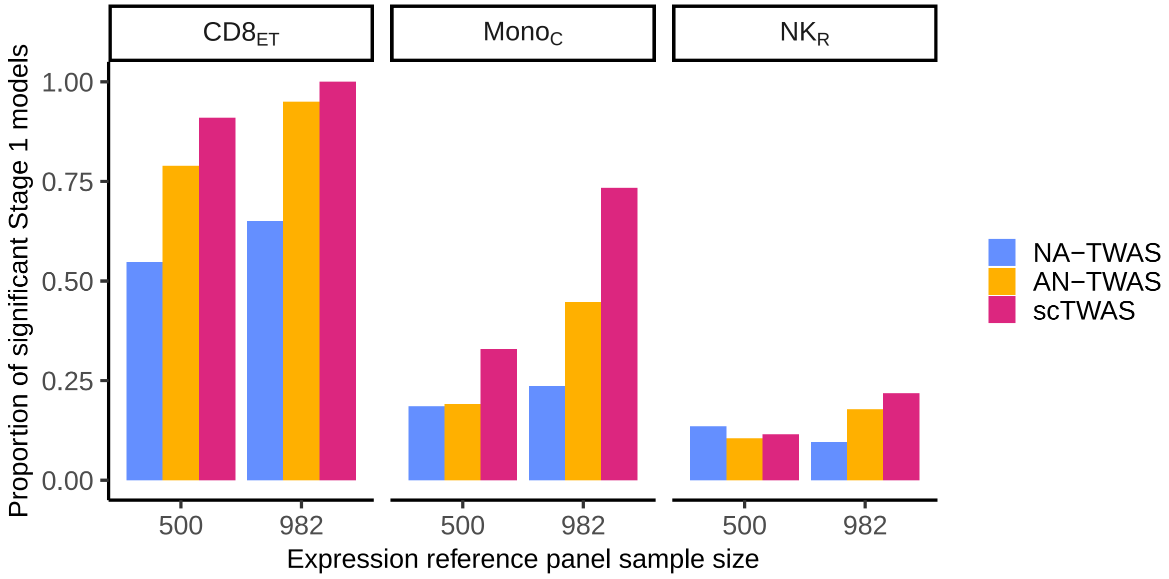

Figure 2. **Proportion of predictive GReX models with nominal p-value < 0.05 in two-sample simulations across $N_{\mathbf{eQTL}} \in \{500, 982\}$.** The nominal p-values are calculated using the same procedure as in Figure 1a.

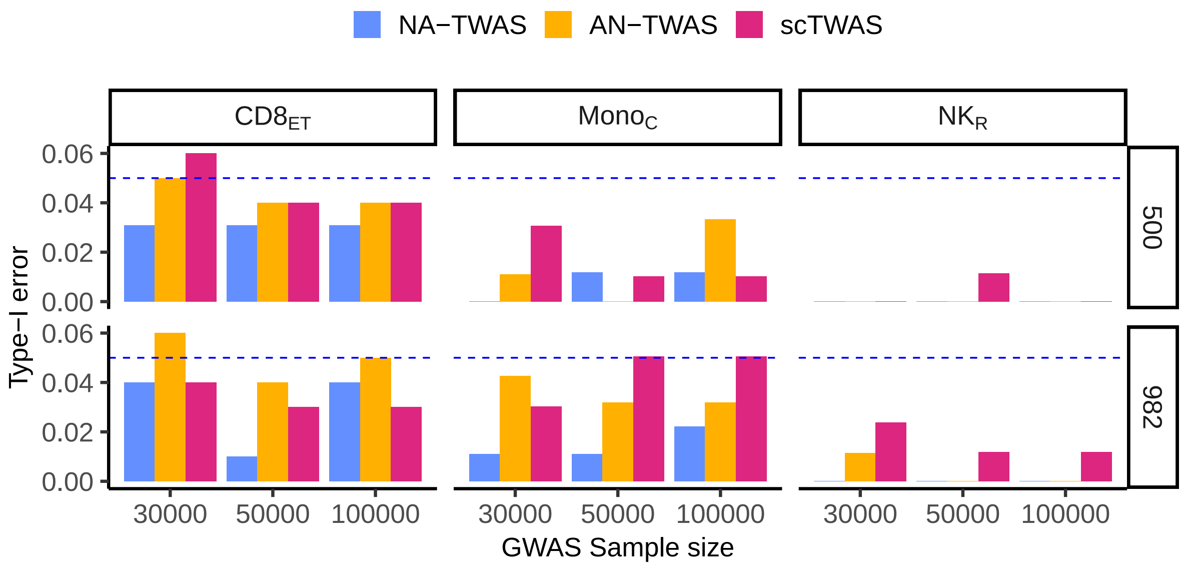

Figure 3. **Empirical type-I error rates of gene-trait association tests in two-sample simulations across $N_{\mathbf{eQTL}} \in \{500, 982\}$ and $N_{\mathbf{GWAS}} \in \{3\times10^4,\ 5\times10^4,\ 10^5\}$.** A significant gene-trait association is counted when both Stage 1 and Stage 2 p-values are smaller than 0.05. The nominal p-values are calculated using the same procedure as in Figures 1a-b.

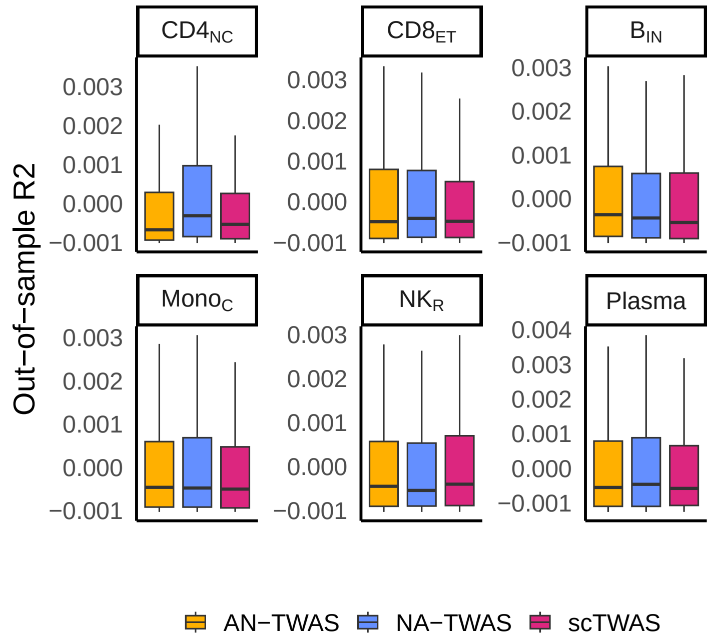

Figure 4. **Out-of-sample R^2 from five-fold cross-validation under the null Stage 1 simulation scenario with $N_{\mathbf{eQTL}} = 982$.** Gene expression was simulated to be independent of *cis*-SNPs, resulting in no true signal in Stage 1. Boxplots display the median (center), the first to the third quartiles (box), and whiskers extending to values within 1.5× the distance between the quartiles, based on 1 000 simulation replicates for each cell type.

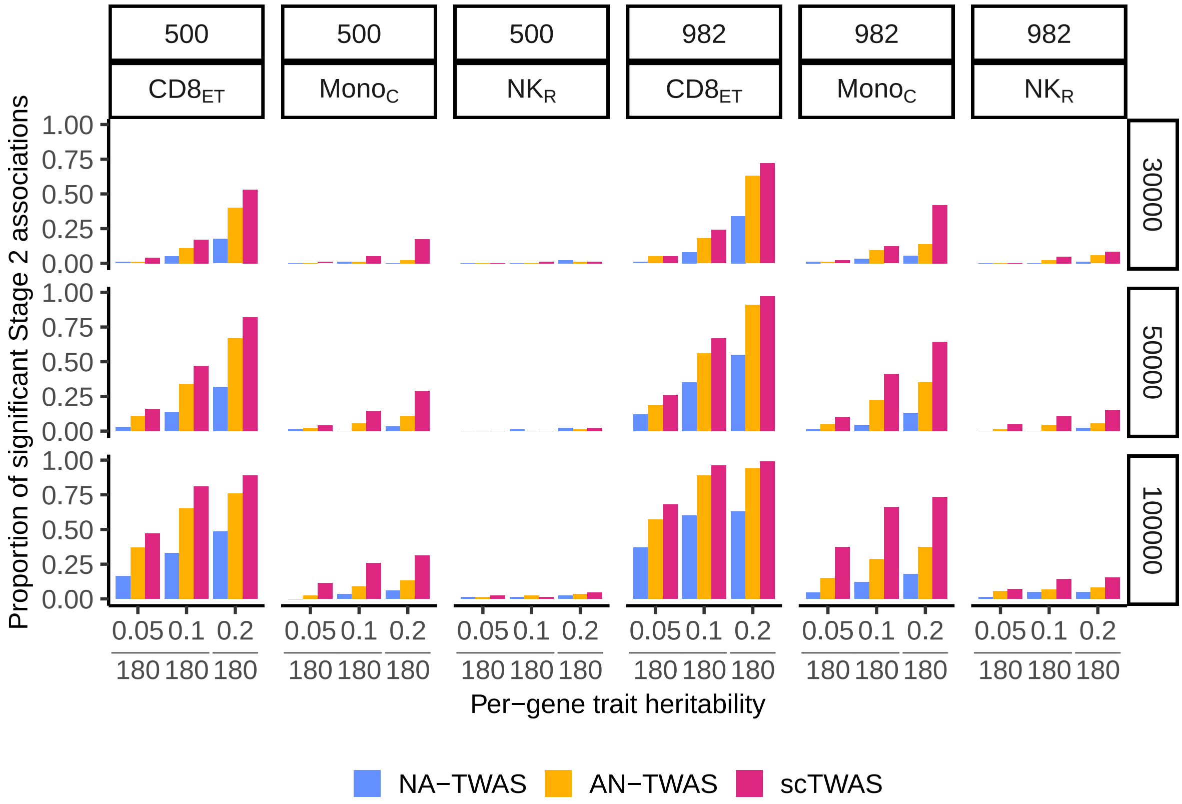

Figure 5. **Empirical power to detect gene-trait associations in two-sample simulations across different per-gene trait heritability** (0.05/180, 0.1/180, 0.2/180)**, expression reference panel sample sizes** (500 **and** 982)**, GWAS sample sizes** (3×10^4, 5×10^4, 10^5)**, and three cell types.** A significant Stage 2 association is counted when both Stage 1 p-value < 0.05 and Stage 2 p-value $< 0.05/M$, where $M = 1234$ for CD8_{ET}, 476 for Mono_{C}, and 122 for NK_{R}. The nominal p-values are calculated using the same procedure as in Figures 1a-b.

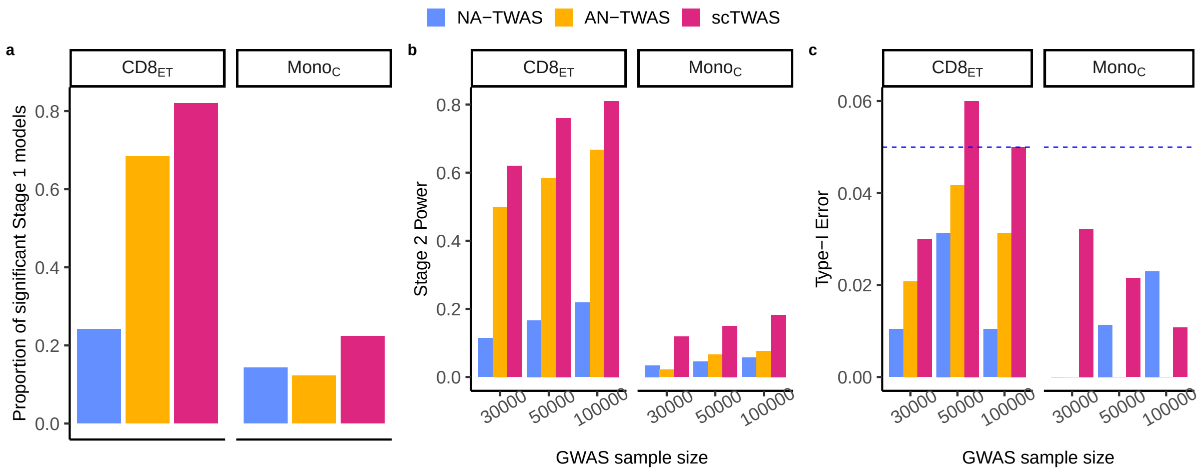

Figure 6. **Simulation results with three causal *cis*-SNPs under a two-sample design. a.** Proportion of predictive GReX models with nominal p-value < 0.05. **b.** Empirical power when $h_g^2 = 0.2/180$. **c.** Empirical type-I error when $h_g^2 = 0$. Stage 1 models were trained with $N_{\text{eQTL}} = 982$. In **a**, the nominal p-values are calculated using the same procedure as in Figure 1a. In **b**, to evaluate power, a significant Stage 2 association is counted when both Stage 1 p-value < 0.05 and Stage 2 p-value $< 0.05/M$, where $M = 1234$ for CD8_{ET} and 476 for Mono_{C}. In **c**, to evaluate type-I error, a significant Stage 2 association is counted when both Stage 1 and 2 p-values < 0.05. In **b-c**, the nominal p-values are calculated using the same procedure as in Figure 1b.

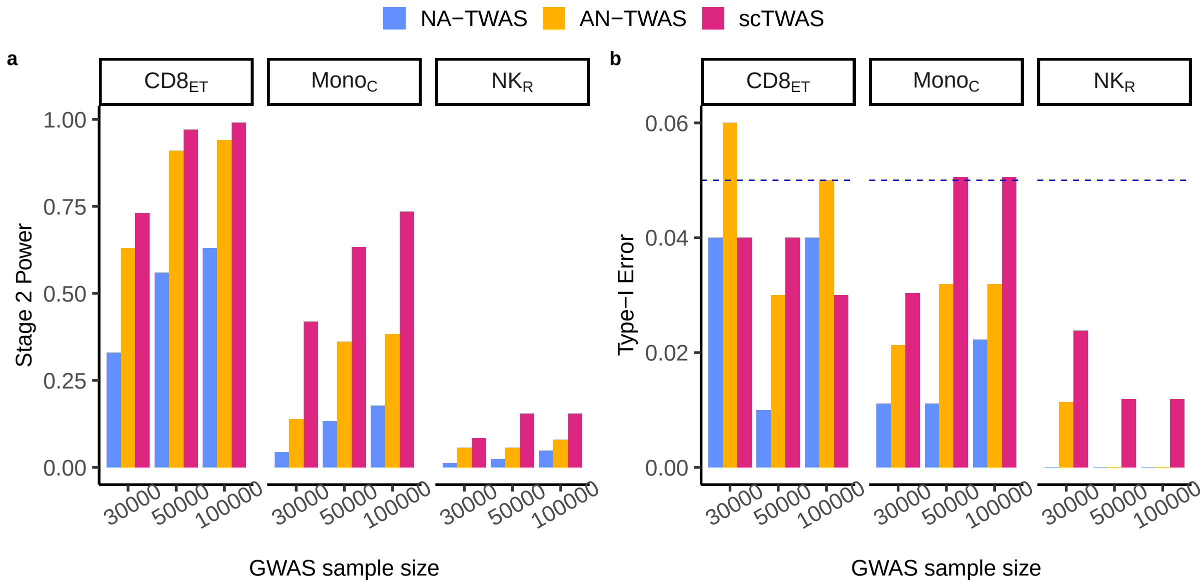

Figure 7. **Simulation results with an out-of-sample LD in Stage 2 and $N_{\text{eQTL}} =$ 982 under a two-sample design. a.** Empirical power when $h_g^2 = 0.2/180$. **b.** Empirical type-I error when $h_g^2 = 0$. Stage 1 models were trained with $N_{\text{eQTL}} = 982$. In **a**, to evaluate power, a significant Stage 2 association is counted when both Stage 1 p-value < 0.05 and Stage 2 p-value $< 0.05/M$, where $M = 1234$ for CD8_{ET}, 476 for Mono_{C}, and 122 for NK_{R}. In **b**, to evaluate type-I error, a significant Stage 2 association is counted when both Stage 1 and 2 p-values < 0.05. The nominal p-values are calculated using the same procedure as in Figures 1a-b.

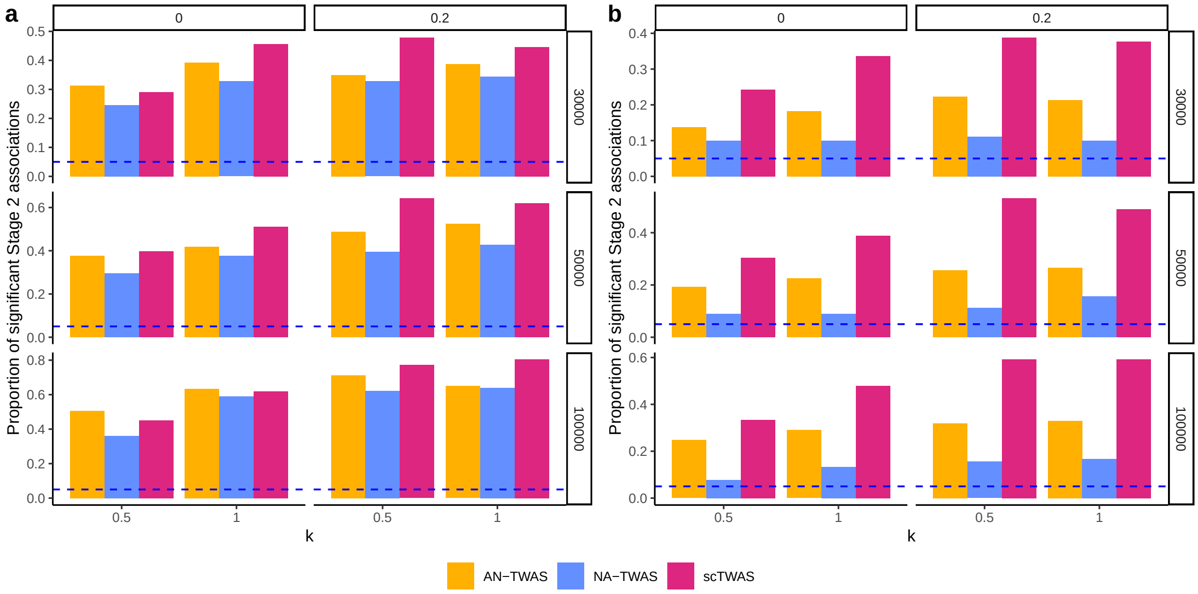

Figure 8. **Simulation results for Mono_C in the presence of horizontal pleiotropy (Supplementary Section 1.3.2) under a two-sample design. a-b.** Empirical type-I error rate when $h_g^2 = 0$ and power when $h_g^2 = 0.2/180$ across k (the strength of pleiotropic effect) and GWAS sample sizes. In **a.**, a significant Stage 2 association was counted as long as Stage 2 test was significant; whereas in **b.**, a significant Stage 2 association was counted when the gene had both a predictive Stage 1 model (with prediction p-value < 0.05) and a significant Stage 2 test. The nominal p-values are calculated using the same procedure as in Figures 1a-b.

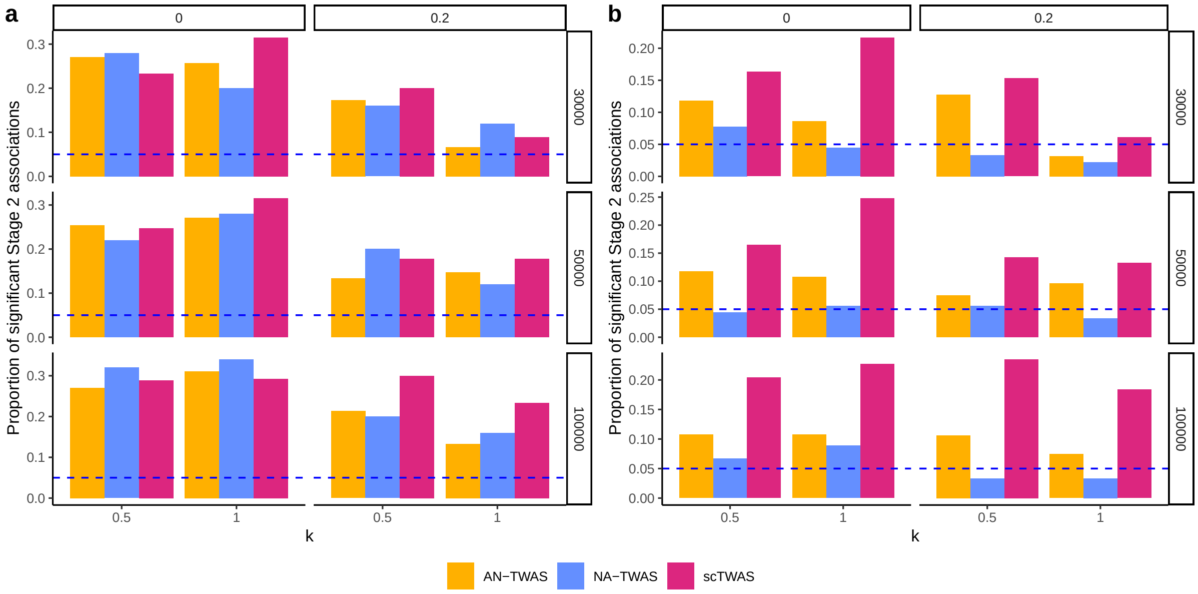

Figure 9. **LDA-Egger results for Mono_C in the presence of horizontal pleiotropy (Supplementary Section 1.3.2) in two-sample simulations. a-b.** Empirical type-I error rate when $h_g^2 = 0$ and power when $h_g^2 = 0.2/180$ across k (the strength of pleiotropic effect) and GWAS sample sizes. In **a.**, a significant Stage 2 association was counted as long as Stage 2 test was significant; whereas in **b.**, a significant Stage 2 association was counted when the gene had both a predictive Stage 1 model (with prediction p-value < 0.05) and a significant Stage 2 test. The nominal p-values are calculated using the same procedure as in Figures 1a-b.

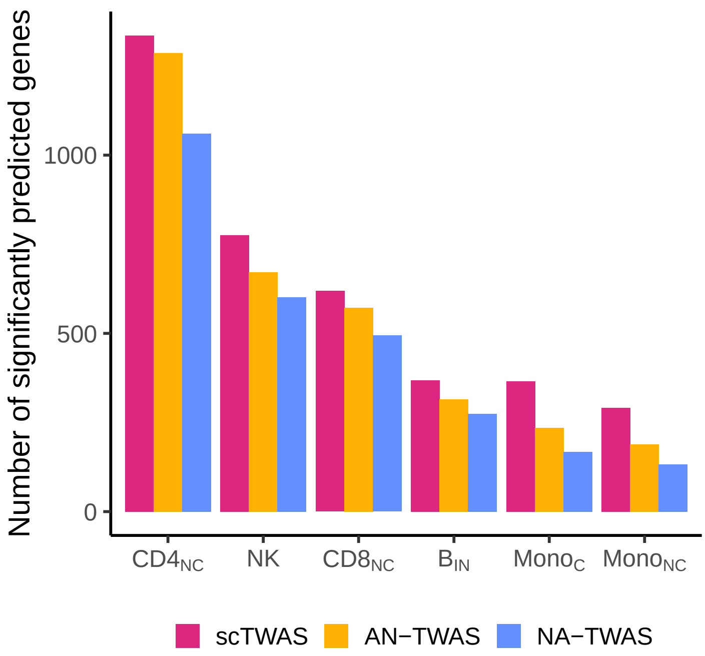

Figure 10. **Number of significantly predicted genes in DICE using GReX models trained on OneK1K scRNA-seq data.** Genes are considered significantly predicted if the Pearson correlation between predicted and observed expression is significant at the nominal level (two-sided Pearson correlation test; $p < 0.05$).

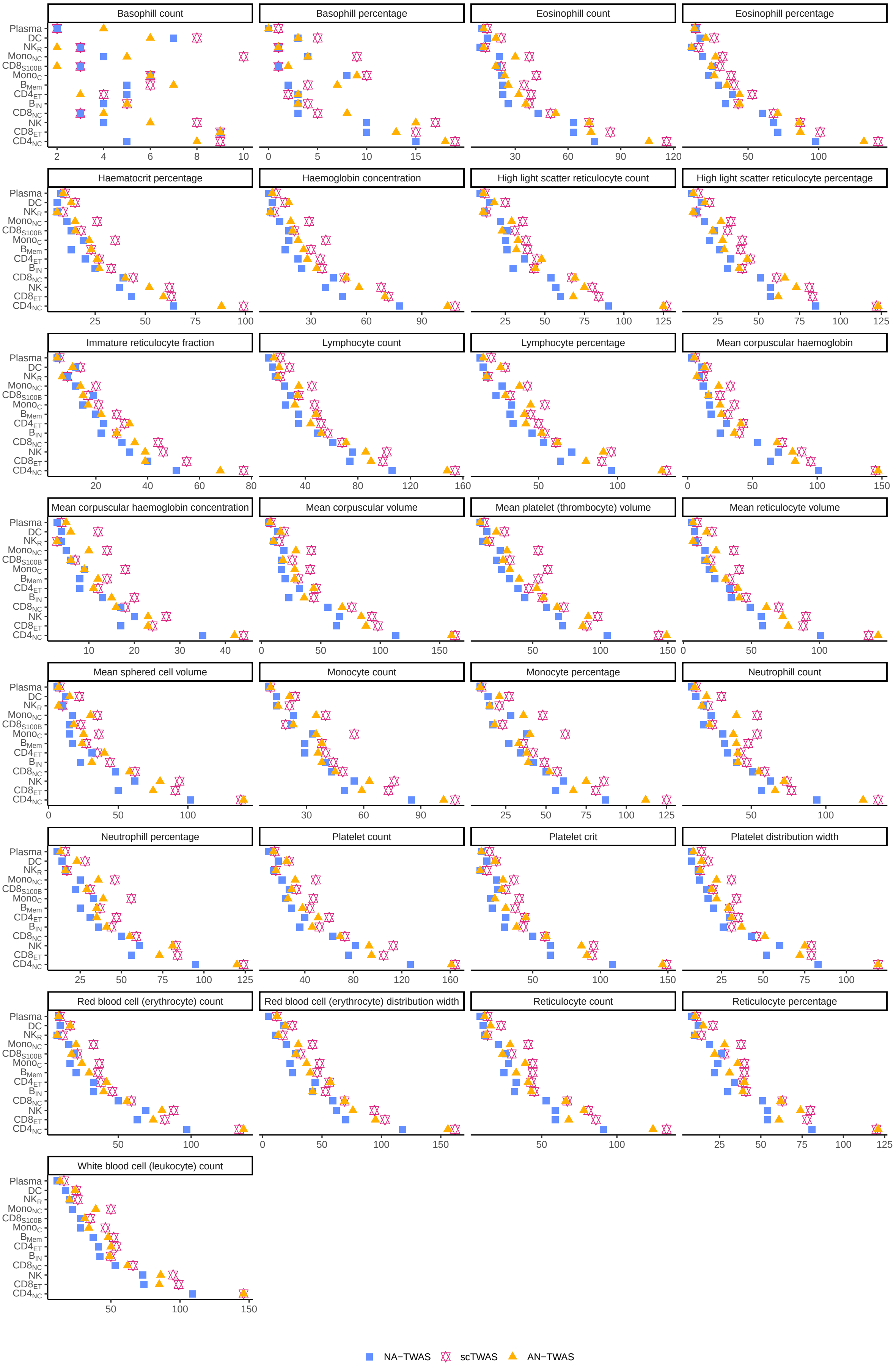

Figure 11. **Number of significant gene-trait associations identified by three methods across 29 blood cell traits.**

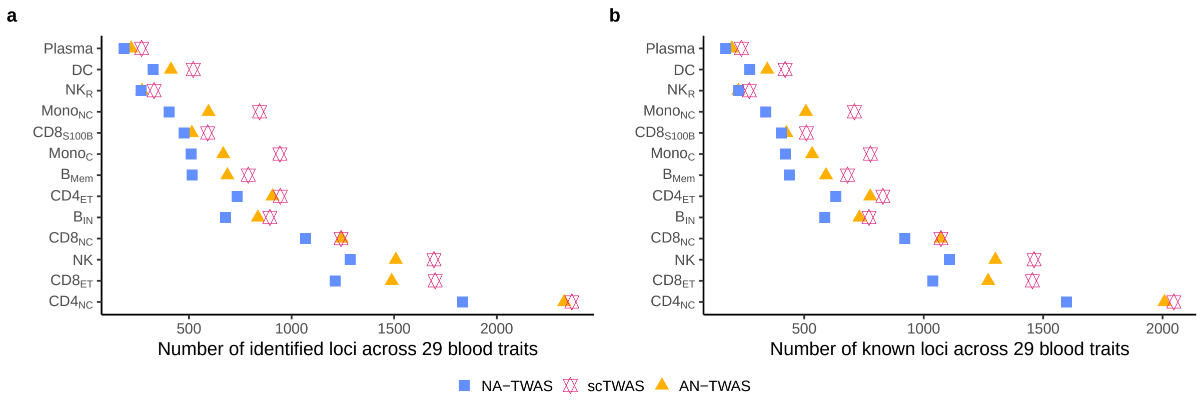

Figure 12. **Results for hematological traits. a.** Total number of TWAS-identified loci and **b.** Number of TWAS loci overlapping known GWAS signals across 29 blood cell traits by three methods.

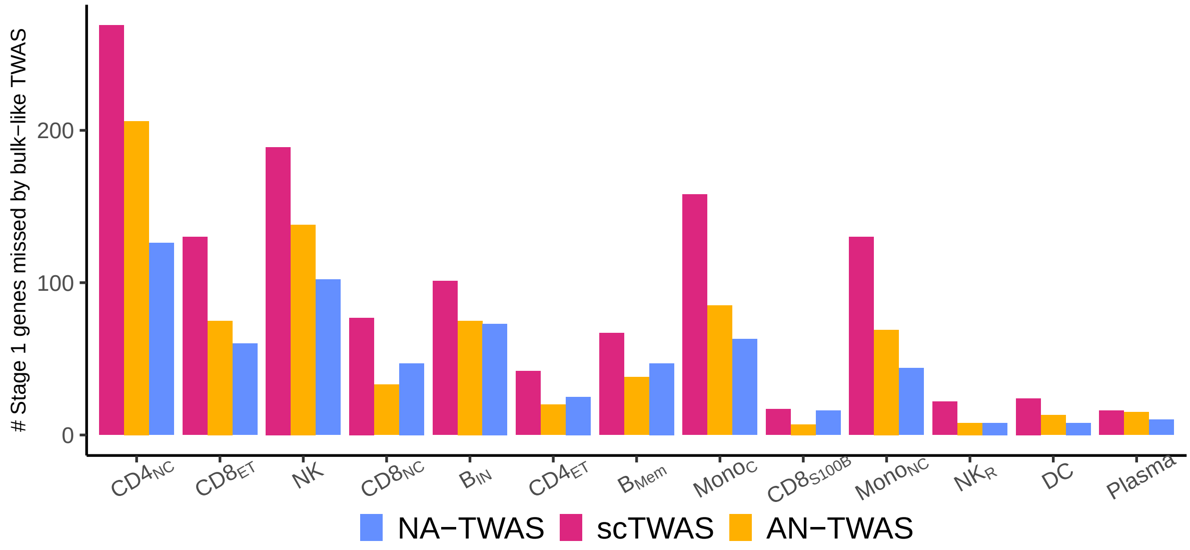

Figure 13. **Number of genes with significantly predictive Stage 1 GReX models in cell-type-specific TWAS that were missed by the bulk-like TWAS across 13 immune cell types.**

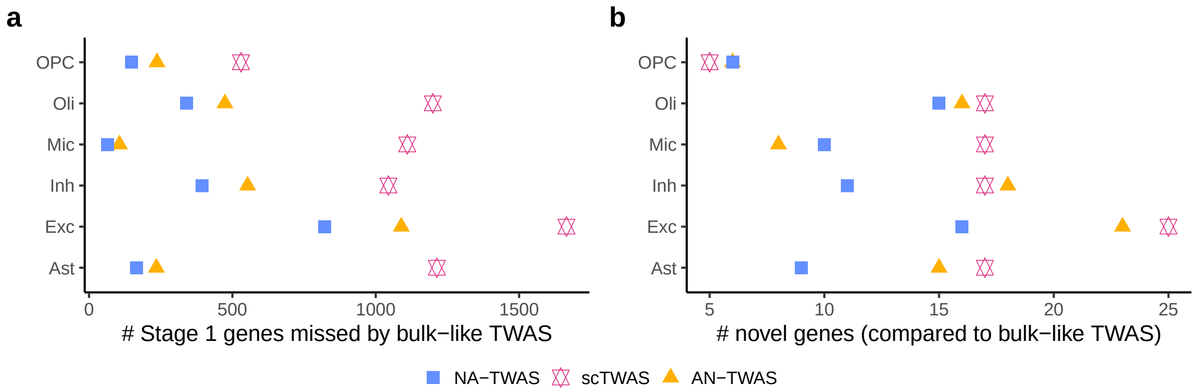

Figure 14. **Comparison between cell-subtype-specific TWAS and bulk-like TWAS in the ROSMAP study. a.** Number of genes with significantly predictive Stage 1 GReX models in brain cell subtypes that were missed by the bulk-like TWAS. **b.** Number of TWAS genes in brain cell subtypes that were missed by the bulk-like TWAS. Subtype counts are aggregated within each major cell type.

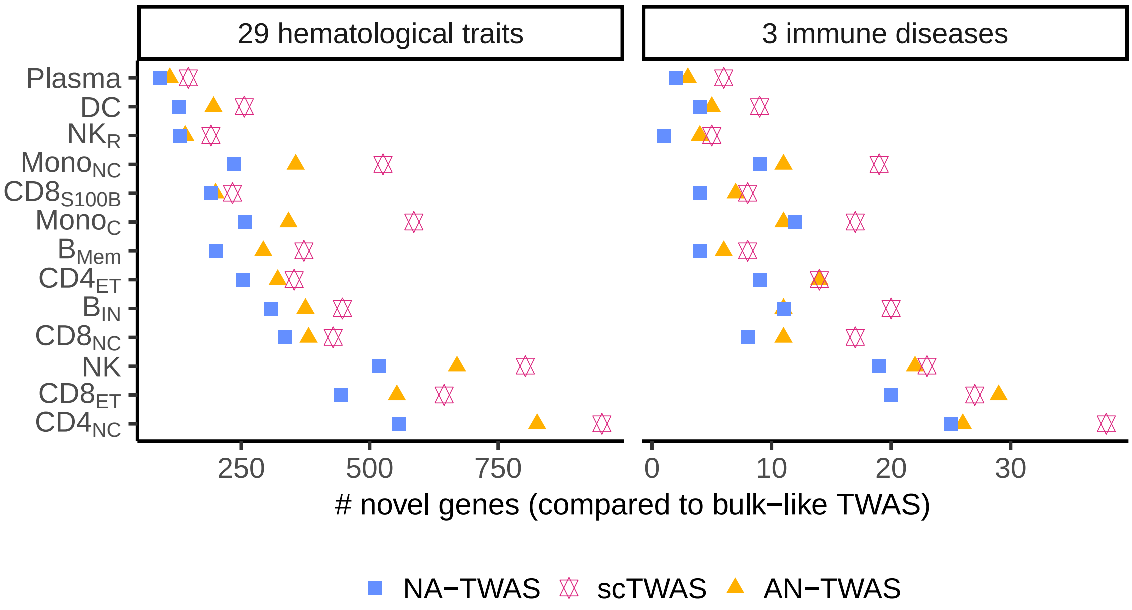

Figure 15. **Number of novel TWAS associations identified by cell-type-specific TWAS that were missed by the bulk-like TWAS, across 29 hematological traits (left) and 3 immune-related diseases (right).**

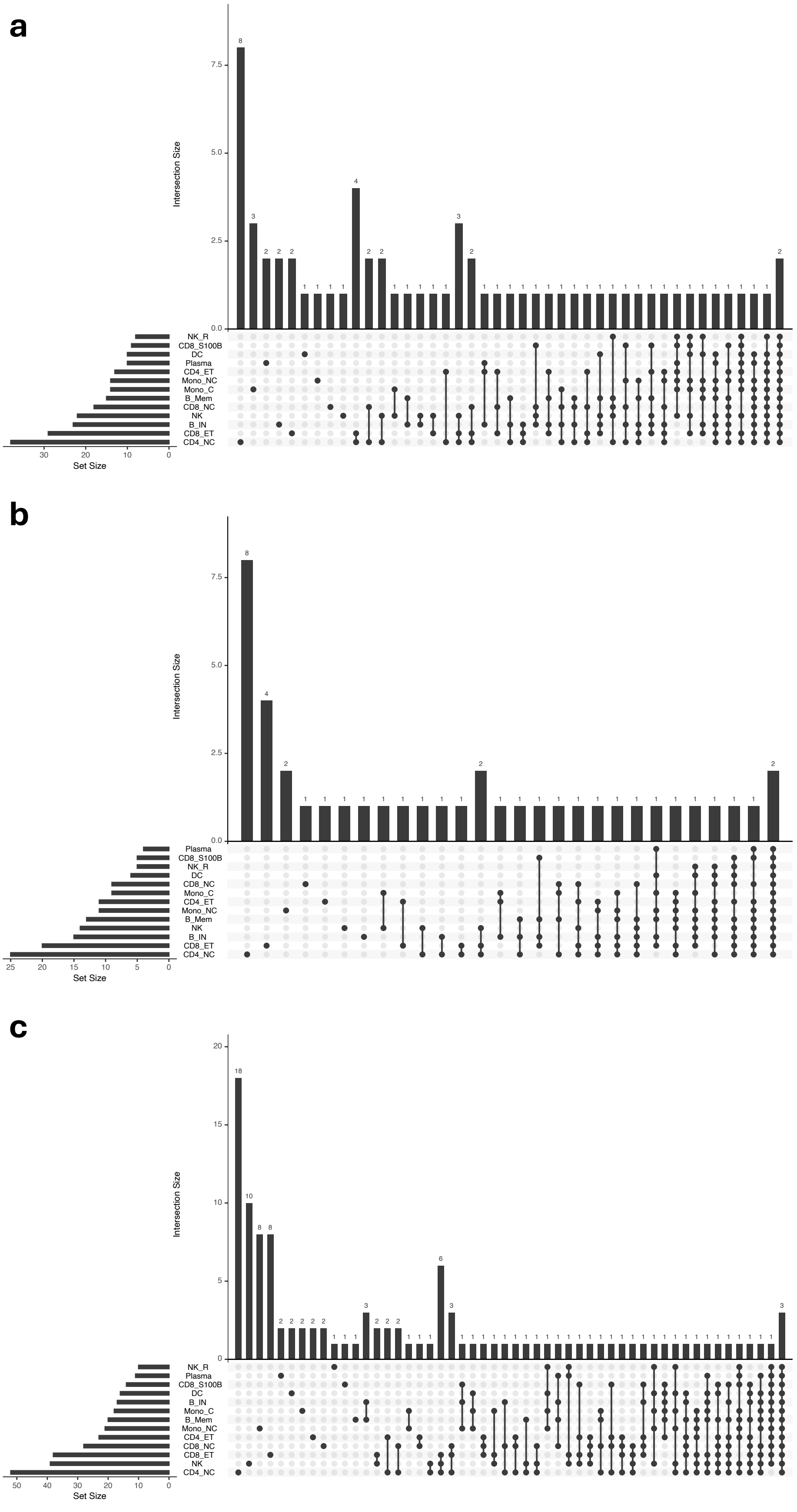

Figure 16. **UpSet plots of scTWAS associations for three immune-related diseases. a.** Rheumatoid arthritis. 21/64 genes are cell-type-specific. **b.** Systemic lupus erythematosus. 20/41 genes are cell-type-specific. **c.** Asthma. 57/111 genes are cell-type-specific.

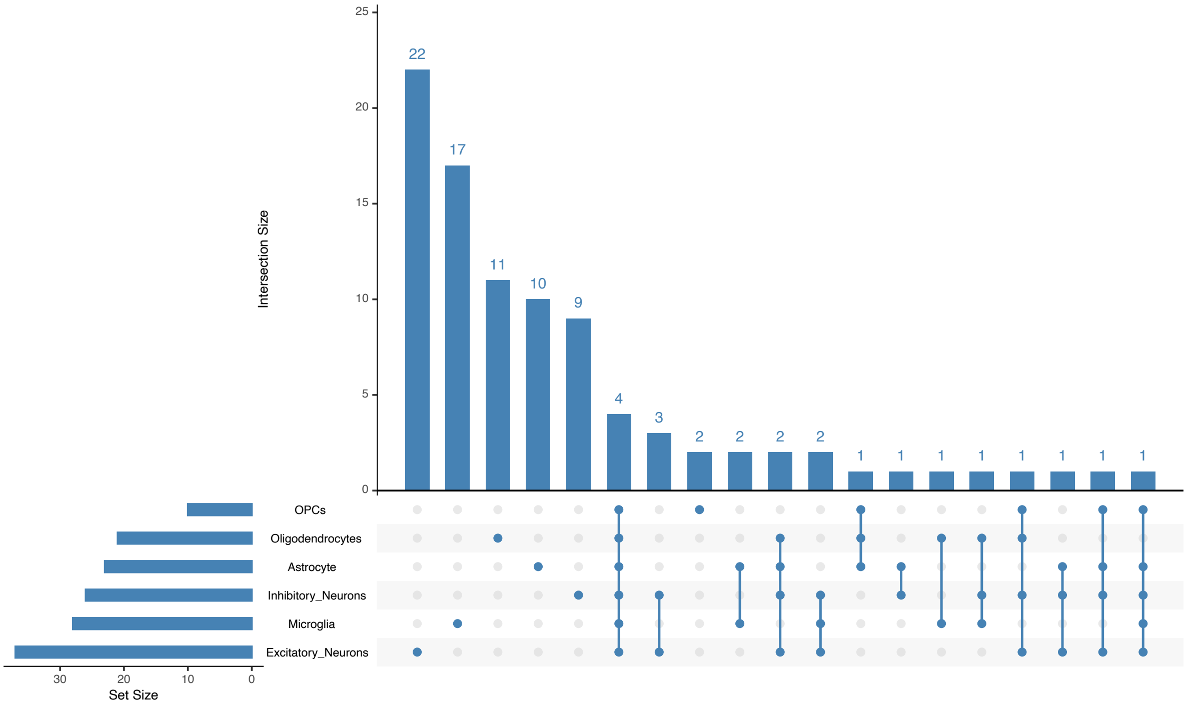

Figure 17. **UpSet plots of scTWAS identified AD genes across all major cell types.** Results are aggregated to the major cell type level.

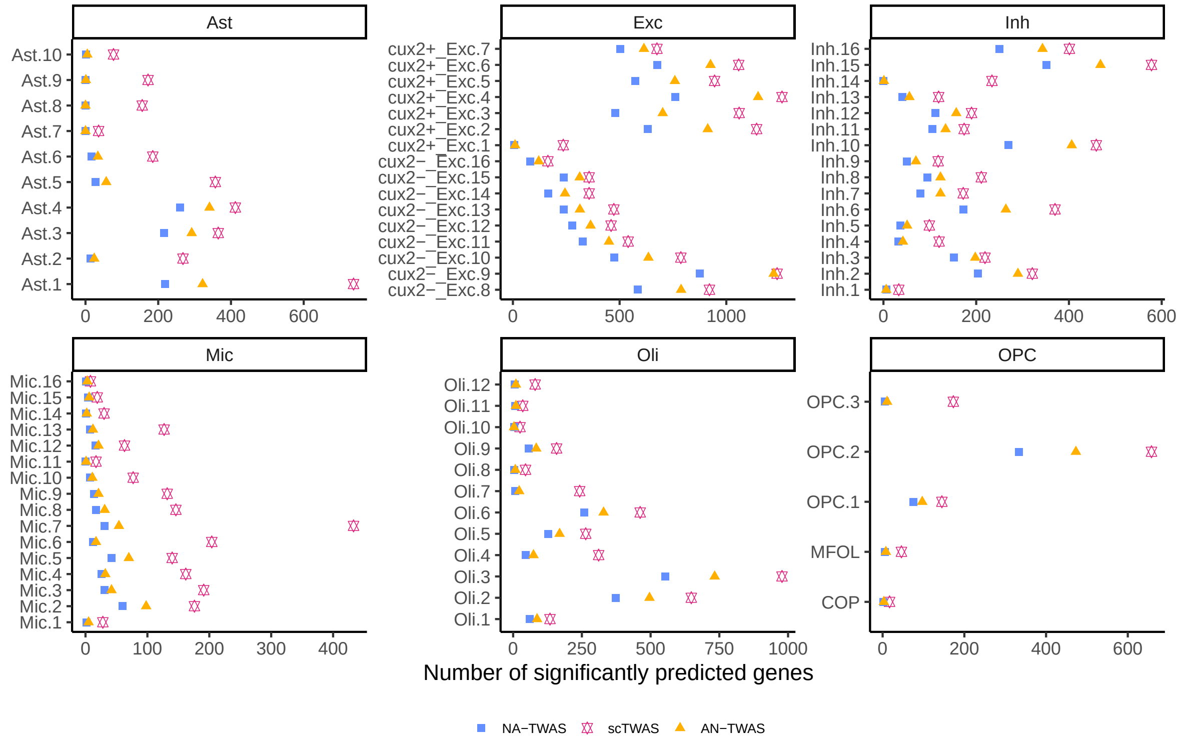

Figure 18. **Number of significant Stage 1 models by scTWAS for 75 brain cell subtypes trained with ROSMAP data on dorsolateral prefrontal cortex [7].**

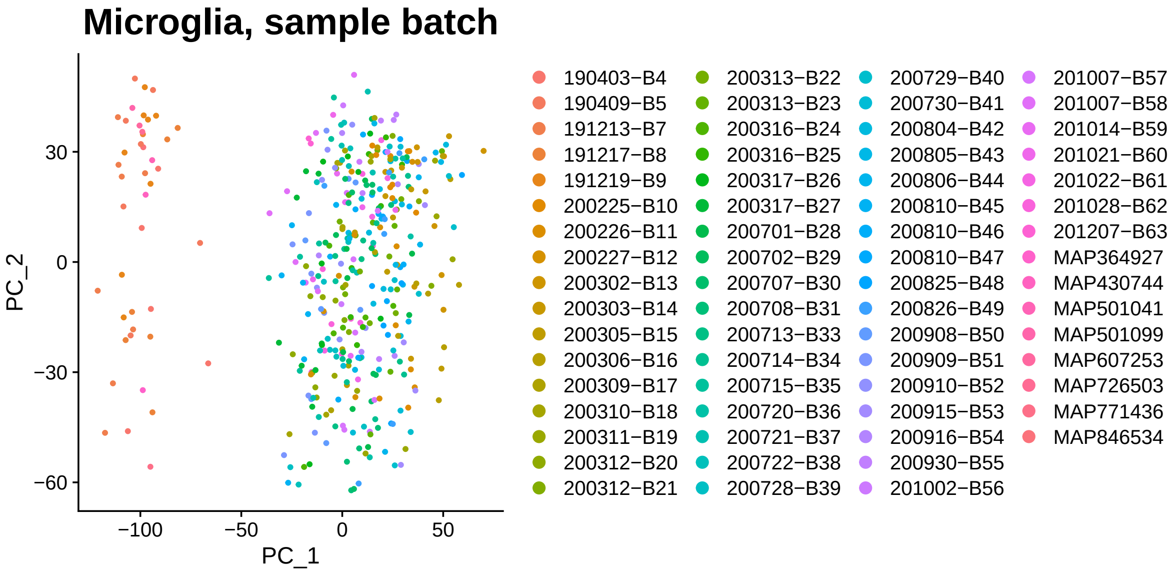

Figure 19. **PCA of pseudo-bulk gene expression in microglia colored by technical batches.**

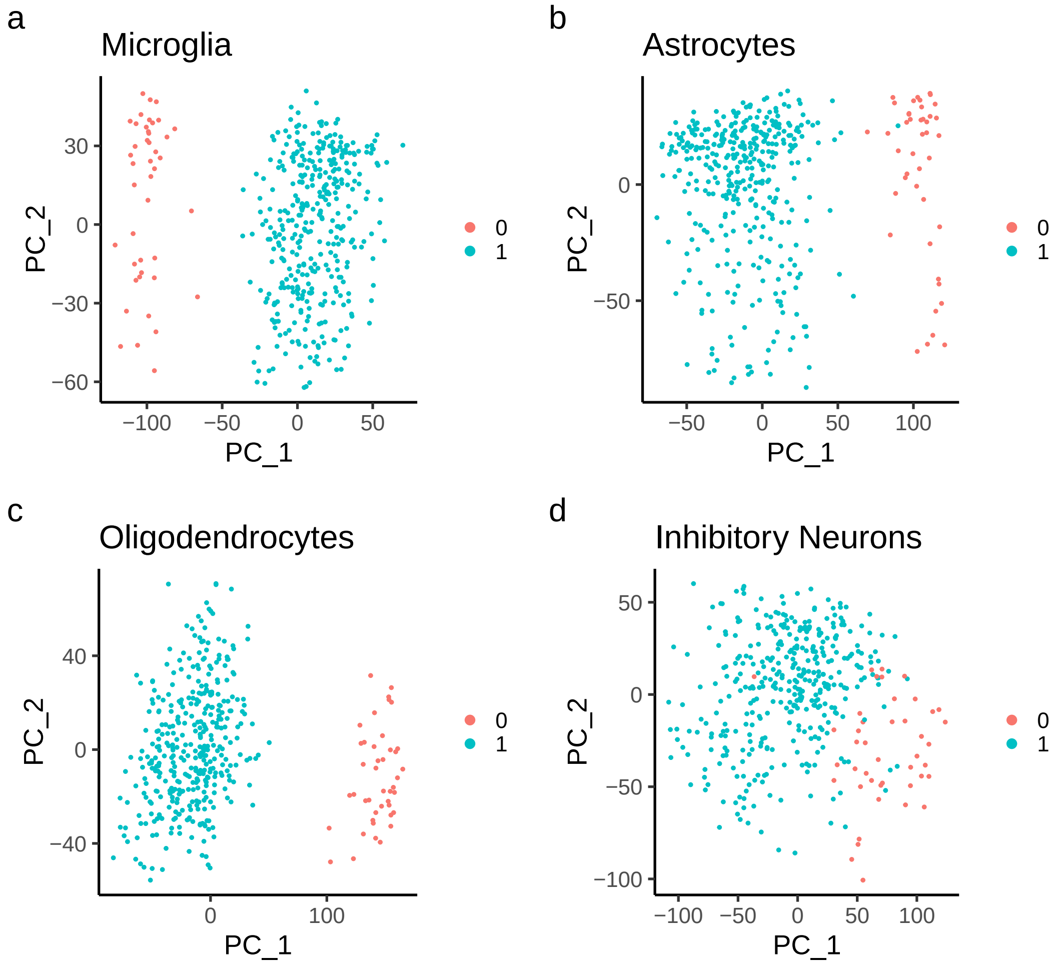

Figure 20. **PCA of pseudo-bulk gene expression colored by the estimated hidden batch effect in four cell types.**

References

[1] Hafemeister, C. & Satija, R. Normalization and variance stabilization of single-cell rna-seq data using regularized negative binomial regression. *Genome biology* **20**, 296 (2019).

[2] Love, M. I., Huber, W. & Anders, S. Moderated estimation of fold change and dispersion for rna-seq data with deseq2. *Genome biology* **15**, 1–21 (2014).

[3] Barfield, R. *et al.* Transcriptome-wide association studies accounting for colocalization using egger regression. *Genetic epidemiology* **42**, 418–433 (2018).

[4] Wainberg, M. *et al.* Opportunities and challenges for transcriptome-wide association studies. *Nature genetics* **51**, 592–599 (2019).

[5] Abe, H., Lin, P., Zhou, D., Ruderfer, D. M. & Gamazon, E. R. Mapping dynamic regulation of gene expression using single-cell transcriptomics and application to complex disease genetics. *Human Genetics and Genomics Advances* **6**, 100397 (2025).

[6] Zeng, L. *et al.* A single-nucleus transcriptome-wide association study implicates novel genes in depression pathogenesis. *Biological Psychiatry* **96**, 34–43 (2024).

[7] Fujita, M. *et al.* Cell subtype-specific effects of genetic variation in the alzheimer's disease brain. *Nature Genetics* **56**, 605–614 (2024).
