# Supplementary material for: scTWAS: a powerful statistical framework for single-cell transcriptome-wide association studies
Source: Nat Commun. 2026 Mar 12;17:3853. doi: 10.1038/s41467-026-70374-7 (PMC13121454; doi:10.1038/s41467-026-70374-7)
Supplement: Supplementary file 2 — Description of Additional Supplementary Files [file 41467_2026_70374_MOESM2_ESM.pdf]

Description of Additional Supplementary Files

Supplementary Data1: A summary of number of individuals, number of cells and number of genes analyzed for each cell (sub)type in OneK1K and ROSMAP datasets.

Supplementary Data2: A full list of genes associated with rheumatoid arthritis identified by scTWAS.

Supplementary Data3: A full list of genes associated with systemic lupus erythematosus identified by scTWAS.

Supplementary Data4: A full list of genes associated with asthma identified by scTWAS.

Supplementary Data5: A full list of genes associated with Alzheimer's disease identified by scTWAS.
