# Supplementary material for: scTWAS: a powerful statistical framework for single-cell transcriptome-wide association studies
Source: Nat Commun. 2026 Mar 12;17:3853. doi: 10.1038/s41467-026-70374-7 (PMC13121454; doi:10.1038/s41467-026-70374-7)
Supplement: Supplementary file 4 — Reporting Summary [file 41467_2026_70374_MOESM4_ESM.pdf]

nature portfolio

Corresponding author(s): Chang Su

Last updated by author(s): Feb 14, 2026

Reporting Summary

Nature Portfolio wishes to improve the reproducibility of the work that we publish. This form provides structure for consistency and transparency in reporting. For further information on Nature Portfolio policies, see our Editorial Policies and the Editorial Policy Checklist.

Statistics

For all statistical analyses, confirm that the following items are present in the figure legend, table legend, main text, or Methods section.

n/a	Confirmed	
☐	☒	The exact sample size (*n*) for each experimental group/condition, given as a discrete number and unit of measurement
☐	☒	A statement on whether measurements were taken from distinct samples or whether the same sample was measured repeatedly
☐	☒	The statistical test(s) used AND whether they are one- or two-sided<br>*Only common tests should be described solely by name; describe more complex techniques in the Methods section.*
☐	☒	A description of all covariates tested
☐	☒	A description of any assumptions or corrections, such as tests of normality and adjustment for multiple comparisons
☐	☒	A full description of the statistical parameters including central tendency (e.g. means) or other basic estimates (e.g. regression coefficient) AND variation (e.g. standard deviation) or associated estimates of uncertainty (e.g. confidence intervals)
☐	☒	For null hypothesis testing, the test statistic (e.g. *F*, *t*, *r*) with confidence intervals, effect sizes, degrees of freedom and *P* value noted<br>*Give P values as exact values whenever suitable.*
☒	☐	For Bayesian analysis, information on the choice of priors and Markov chain Monte Carlo settings
☒	☐	For hierarchical and complex designs, identification of the appropriate level for tests and full reporting of outcomes
☐	☒	Estimates of effect sizes (e.g. Cohen's *d*, Pearson's *r*), indicating how they were calculated

Our web collection on statistics for biologists contains articles on many of the points above.

Software and code

Policy information about availability of computer code

Data collection	No data was collected for the purpose of this study.
Data analysis	R package that implements scTWAS is publicly available at https://github.com/ZhaotongL/scTWAS.<br>Code to reproduce the analysis in the paper is publicly available at https://github.com/ZhaotongL/scTWAS_paper.<br>Other publicly available tools used in this study:<br>FUSION (https://github.com/gusevlab/fusion_twas)<br>Seurat v5.0.2 (https://satijalab.org/seurat/)<br>PLINK 2.0 (https://www.cog-genomics.org/plink/2.0/)<br>gprofiler2 v0.2.3 (https://cran.r-project.org/web/packages/gprofiler2/index.html)

For manuscripts utilizing custom algorithms or software that are central to the research but not yet described in published literature, software must be made available to editors and reviewers. We strongly encourage code deposition in a community repository (e.g. GitHub). See the Nature Portfolio guidelines for submitting code & software for further information.

Data

Policy information about availability of data

All manuscripts must include a data availability statement. This statement should provide the following information, where applicable:

- Accession codes, unique identifiers, or web links for publicly available datasets
- A description of any restrictions on data availability
- For clinical datasets or third party data, please ensure that the statement adheres to our policy

The GReX prediction models for immune and brain cell types generated in this study have been deposited in Figshare at [https://doi.org/10.6084/m9.figshare.31329184] and [https://doi.org/10.6084/m9.figshare.31329139] \cite{wgt_figshare_OneK1K, wgt_figshare_ROSMAP}.
The availability for datasets used to train or validate GReX models is as follows:
OneK1K genotype and scRNA-seq data are publicly available from the OneK1K study \cite{yazar2022single}.
Genotype and gene expression data of immune cell types from the DICE project \cite{schmiedel2018impact} are available under controlled access in dbGap under accession code phs001703.v5.p1 [https://www.ncbi.nlm.nih.gov/projects/gap/cgi-bin/study.cgi?study_id=phs001703.v5.p1], access can be obtained by requesting Authorized Access on dbGap. WGS and snRNA-seq data from the ROSMAP study are available on Synapse under controlled access with accession codes syn11724057 [https://www.synapse.org/Synapse:syn11724057], syn53366818 [https://www.synapse.org/Synapse:syn53366818], and syn52293417 [https://www.synapse.org/Synapse:syn52293417], access can be obtained by submitting a Data Use Certificate to Synapse.

Research involving human participants, their data, or biological material

Policy information about studies with human participants or human data. See also policy information about sex, gender (identity/presentation), and sexual orientation and race, ethnicity and racism.

Reporting on sex and gender	No sex- or gender-based analyses were conducted
Reporting on race, ethnicity, or other socially relevant groupings	ROSMAP and OneK1K data were mainly European.
Population characteristics	For OneK1K, the age ranges from 19 to 97, with a median of 67; It contains 565 females and 417 males.<br>For ROSMAP, the age ranges from 67 to 90+ (censored) with a median of 90; and it contains 288 females and 136 males.
Recruitment	For OneK1K, see https://www.science.org/doi/10.1126/science.abf3041;<br>For ROSMAP, see https://www.nature.com/articles/sdata2018142.
Ethics oversight	N/A

Note that full information on the approval of the study protocol must also be provided in the manuscript.

Field-specific reporting

Please select the one below that is the best fit for your research. If you are not sure, read the appropriate sections before making your selection.

☒ Life sciences ☐ Behavioural & social sciences ☐ Ecological, evolutionary & environmental sciences

For a reference copy of the document with all sections, see nature.com/documents/nr-reporting-summary-flat.pdf

Life sciences study design

All studies must disclose on these points even when the disclosure is negative.

Sample size	We used OneK1K, ROSMAP and GWAS datasets that had pre-determined sample sizes.
Data exclusions	We didn't exclude individual from any of the datasets.
Replication	We tested the model on independent datasets, as described in Section 4.5.1.
Randomization	Not applicable.
Blinding	Not applicable.

Reporting for specific materials, systems and methods

We require information from authors about some types of materials, experimental systems and methods used in many studies. Here, indicate whether each material, system or method listed is relevant to your study. If you are not sure if a list item applies to your research, read the appropriate section before selecting a response.

Materials & experimental systems

n/a	Involved in the study
☒	☐ Antibodies
☒	☐ Eukaryotic cell lines
☒	☐ Palaeontology and archaeology
☒	☐ Animals and other organisms
☒	☐ Clinical data
☒	☐ Dual use research of concern
☒	☐ Plants

Methods

n/a	Involved in the study
☒	☐ ChIP-seq
☒	☐ Flow cytometry
☒	☐ MRI-based neuroimaging

Plants

Seed stocks	*Report on the source of all seed stocks or other plant material used. If applicable, state the seed stock centre and catalogue number. If plant specimens were collected from the field, describe the collection location, date and sampling procedures.*
Novel plant genotypes	*Describe the methods by which all novel plant genotypes were produced. This includes those generated by transgenic approaches, gene editing, chemical/radiation-based mutagenesis and hybridization. For transgenic lines, describe the transformation method, the number of independent lines analyzed and the generation upon which experiments were performed. For gene-edited lines, describe the editor used, the endogenous sequence targeted for editing, the targeting guide RNA sequence (if applicable) and how the editor was applied.*
Authentication	*Describe any authentication procedures for each seed stock used or novel genotype generated. Describe any experiments used to assess the effect of a mutation and, where applicable, how potential secondary effects (e.g. second site T-DNA insertions, mosiacism, off-target gene editing) were examined.*
